# Reproductive Dynamics and Potential Annual Fecundity of South Pacific Albacore Tuna (*Thunnus alalunga*)

**DOI:** 10.1371/journal.pone.0060577

**Published:** 2013-04-02

**Authors:** Jessica H. Farley, Ashley J. Williams, Simon D. Hoyle, Campbell R. Davies, Simon J. Nicol

**Affiliations:** 1 Wealth from Oceans Flagship, CSIRO Marine and Atmospheric Research, Hobart, Tasmania, Australia; 2 Oceanic Fisheries Programme, Secretariat of the Pacific Community, Noumea, New Caledonia; University of Bologna, Italy

## Abstract

The reproductive biology of albacore tuna, *Thunnus alalunga,* in the South Pacific Ocean was investigated with samples collected during broad-scale sampling between 2006 and 2011. Histology was done in a single laboratory according to standard protocols and the data analysed using generalized linear mixed-effects models. The sex ratio of albacore was female biased for fish smaller than approximately 60 cm *FL* and between 85 and 95 cm, and progressively more male biased above 95 cm *FL.* Spawning activity was synchronised across the region between 10°S and 25°S during the austral spring and summer where sea surface temperatures were ≥24 °C. The average gonad index varied among regions, with fish in easterly longitudes having heavier gonads for their size than fish in westerly longitudes. Albacore, while capable of spawning daily, on average spawn every 1.3 days during the peak spawning months of October to December. Spawning occurs around midnight and the early hours of the morning. Regional variation in spawning frequency and batch fecundity were not significant. The proportion of active females and the spawning fraction increased with length and age, and mature small and young fish were less active at either end of the spawning season than larger, older fish. Batch fecundity estimates ranged from 0.26 to 2.83 million oocytes with a mean relative batch fecundity of 64.4 oocytes per gram of body weight. Predicted batch fecundity and potential annual fecundity increased with both length and age. This extensive set of reproductive parameter estimates provides many of the first quantitative estimates for this population and will substantially improve the quality of biological inputs to the stock assessment for South Pacific albacore.

## Introduction

Albacore tuna, *Thunnus alalunga,* are widely distributed in tropical, sub-tropical and temperate zones between approximately 10–50°N and 5–45°S worldwide [1]. Industrial-scale fishing for albacore began in the 1950's and current catches are estimated at 6% of the global tuna catch [2], [3]. The species is currently managed as six separate stocks that occur in the north and south Pacific, the north and south Atlantic, the Indian Ocean and Mediterranean Sea [4], [5]. Assessments of the status of albacore stocks currently range from overexploited in the North Atlantic Ocean through to moderately exploited in the South Pacific Ocean [6], [7], [8], [9], [10]. Although the stock in the South Pacific is not considered to be overfished, the fisheries in this region have expanded considerably in the last two decades and consequently the catch from the South Pacific Ocean has increased from 34,126 mt in 1990 to 88,919 mt in 2010 [11].

The life cycle and migration routes of albacore in the South Pacific are poorly understood. Spawning is thought to occur predominantly between 10–25°S in the western and central regions to about 140°W, where surface water temperatures exceed 24 °C [12], [13], [14]. By age 1 year (∼45–50 cm fork length; *FL*), juveniles move south and recruit to the New Zealand surface fisheries and the U.S. troll fishery in the sub-tropical convergence zone (to approximately 130°W) [15]. These fisheries predominantly catch juveniles and sub-adults up to ∼80–90 cm *FL* from December to April. It has been suggested that juveniles remain south of 30°S and do not return to the sub-tropics or tropics until they mature [16], [17], [18]. Catch rates of albacore in the sub-tropics usually peak in December–January and May–July [19], consistent with an annual north-south migration of albacore.

Despite the long history of albacore fisheries and general knowledge of its life-history, there are few quantitative estimates of size- or age-specific reproductive parameters for albacore in the South Pacific Ocean. A common assumption in many fishery stock assessments is that population dynamics are uniform across the entire distribution of the stock, or that fish mix sufficiently rapidly that any substantial variation is not important for assessment purposes [20]. Currently, many of the estimates of reproductive parameters included in the stock assessment model for albacore in the South Pacific Ocean [10] are derived from studies of other stocks or tuna species [21]. Sensitivity analyses using the stock assessment demonstrate that changing these parameter estimates by a small amount can substantially affect the values of reference points that are important for management advice, such as the size and level of depletion of the spawning biomass [21]. Consequently, possible differences in population biology among stocks of albacore in the Pacific and other oceans may have large implications for the South Pacific stock assessment, the fishery and robust management advice. Here, the reproduction and spawning dynamics of albacore in the South Pacific are examined including (1) sex ratio, (2) reproductive status, (3) spatio-temporal aspects of spawning, (4) spawning periodicity and frequency, (5) batch fecundity, and (6) potential annual fecundity. Spatial variation in these parameters is also examined. A comprehensive description of the maturity schedule for South Pacific albacore is provided in [22].

## Methods

### Ethical statement

Ethical approval was not required for this study, as all fish were collected as part of routine fishing procedures. No samples were collected by the authors. All samples in this study originated from commercial or recreational fisheries (New Zealand commercial Albacore Fishery, Western Central Pacific Ocean commercial longline fishery, and Australian commercial Eastern Tuna and Billfish Fishery [ETBF] and recreational fishery) and were already dead when provided to the sampler. Fish were sacrificed by the commercial or recreational fisher at sea using standard fisheries practices (most fish were dead when landed). Permission was granted to use samples from all fish. All samples were donated.

No field permits were required to collect any samples from any location, as all samples originated from commercial and recreational catch. Albacore tuna are not a protected species in any ocean.

### Sample collection

**Figure 1:**
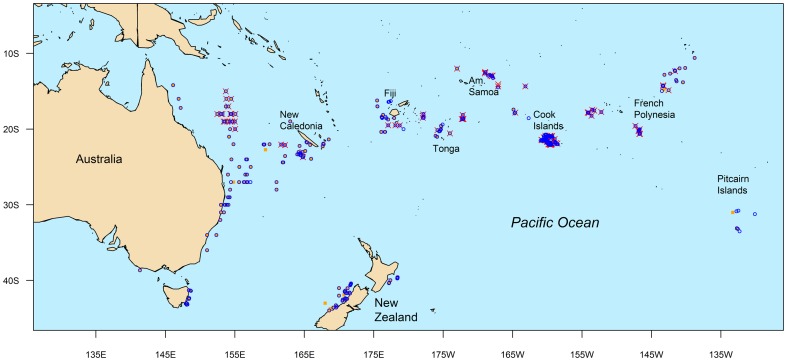
Map indicating locations where samples were collected from South Pacific albacore. Localities are shown for active female (red crosses), inactive or immature female (orange squares) and male (blue circles).

Gonads were collected from albacore caught across the southwest Pacific Ocean between November 2006 and January 2011 (Fig. 1; Table 1). The majority of samples were collected from fish caught in commercial troll and longline fisheries in Australia, New Zealand, New Caledonia, Fiji, Tonga, American Samoa, Cook Islands, French Polynesia, and in a region south of the Pitcairn Islands. Samples from Australia were collected in port from fish caught by the domestic longline fishery along almost the entire mainland coast between 14°S and 37°S. Additional samples were collected from small fish caught by recreational fishers in southern Australia between 37°S and 44°S. Samples from New Zealand were collected either in port from the domestic troll fishery or at sea during chartered tagging operations. Samples collected from all other regions were collected either by observers on longline fishing vessels or directly by the fishing crew of longline fishing vessels.

**Table 1:** Number of South Pacific albacore gonads sampled by region.

EEZ	Female	Male	Total
American Samoa	163	241	404
Australia	641	855	1496
Cook Islands	55	131	186
Fiji	41	160	201
French Polynesia	165	198	363
International Waters 1	21	9	30
International Waters 2	3	6	9
New Caledonia	104	122	226
New Zealand	264	185	449
Tonga	70	63	133
Total	1527	1970	3497

International Waters 1 refers to the waters between the Australian and New Caledonian Exclusive Economic Zones (EEZ), International Waters 2 refers to the waters south of the Pitcairn Islands.

All fish were measured to the nearest cm (*FL*) apart from 87 sampled in Fiji which were measured to the nearest (lower) 5 cm, and 9 from south of Pitcairn Island for which only weights were measured. Whole weight (*W*) was measured to the nearest 0.1 kg for most fish sampled in Australia and south of Pitcairn Island. The *FL* of fish sampled south of Pitcairn Island was estimated from the length-weight relationship [23]. Gonads were removed and either frozen immediately or sent to the laboratory fresh. Sex was identified (microscopically if necessary) in the laboratory and gonads were trimmed of fat and weighed (*GW*) to the nearest g if sampled whole (n = 1695). Gonad index (*GI*) was calculated as: *GI  =  GW/FL^3^ × 10^4^.* A core subsample was taken from each gonad and fixed in 10% buffered formalin. Estimates of annual age were obtained from counts of annual increments in otoliths [24] for 526 females sampled between January 2009 and December 2010. Otoliths were selected for age estimation based on sampling location and *FL* with the aim of estimating age for the full size range of females caught in each region. Sea surface temperatures (SST) at sampling locations were obtained from the Group for High Resolution Sea Surface Temperature (GHRSST) microwave/infrared global composite dataset [25] using the spatial dynamics ocean data explorer (SDODE) interface [26].

### Female histological classification

**Figure 2:**
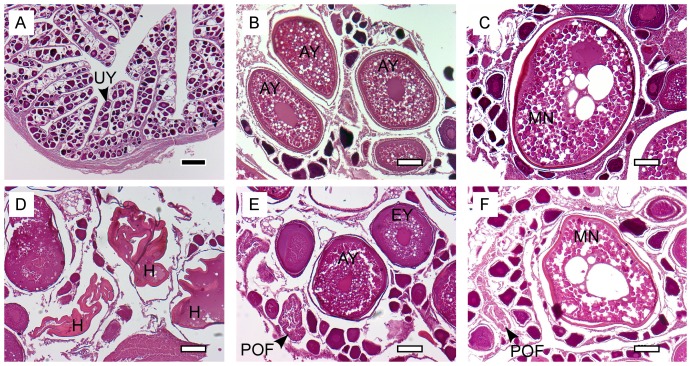
Histological sections of ovaries showing examples of development classes, oocyte stages and postovulatory follicles (POF). (A) unyolked oocytes in an immature ovary, (B) advanced yolked oocytes in a spawning capable ovary, (C) migratory nucleus oocyte in a spawning ovary, (D) hydrated oocytes in a spawning ovary, (E) <12 hour POF in a spawning ovary, (F) >12 hour POF in a spawning ovary. UY  =  unyolked, EY  =  early yolked, AY  =  advanced yolked, MN  =  migratory nucleus, H  =  hydrated. The scale bars are 200 µm (black) and 100 µm (white).

**Figure 3:**
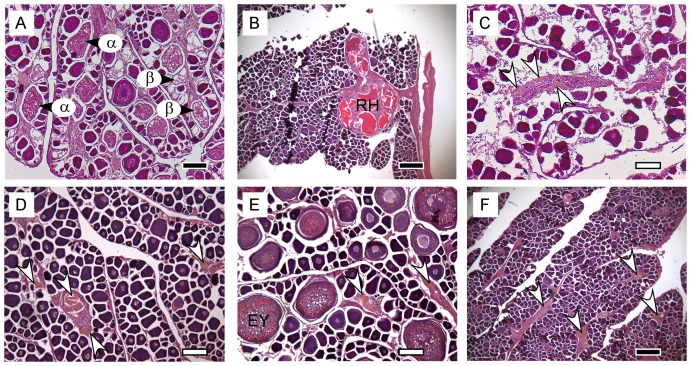
Histological sections of ovaries showing examples of development classes and maturity markers. (A) alpha and beta atresia in a regressed 1 ovary, (B) residual (unovulated) hydrated oocytes in a regenerating ovary, (C–F) late stage atresia (brown bodies; arrows). α  =  alpha atresia, β  =  beta atresia, RH  =  residual hydrated oocyte, EY  =  early yolked oocyte. The scale bars are 200 µm (black) and 100 µm (white).

Ovaries from females ≥70 cm *FL* (n = 1219) were selected for histological analysis as this size was well below the minimum size at maturity (∼80 cm) previously estimated for albacore [27], [28]. Tissue samples were embedded in paraffin and standard histological sections prepared (cut to 8 µm and stained with Harris' haematoxylin and eosin). Ovaries were classified using criteria similar to those developed for yellowfin tuna *Thunnus albacares* [29], [30], bigeye tuna *Thunnus obesus* [31], and southern bluefin tuna *Thunnus maccoyii* [32] based on:

1. The most advanced group of oocytes (MAGO) present (Fig. 2a–d): unyolked, early yolked, advanced yolked, migratory nucleus, and hydrated.
2. The presence and approximate age of postovulatory follicles (POFs) (Fig. 2e–f): absent, new (stage 1), <12 hours (stage 2), 12–24 hours (stage 3). The ages of POFs were estimated based on criteria developed for northern anchovy (*Engraulis mordax*), and bigeye and yellowfin tuna [29], [33], [34].
3. The level of alpha atresia of advanced yolked oocytes (Fig. 3a): absent, <50%, ≥50, 100%.
4. The presence/absence of beta stage atresia of advanced yolked oocytes (Fig. 3a).
5. The presence/absence of maturity markers indicating previous ovary development. The maturity markers used were (i) residual (unovulated) hydrated oocytes which may be encapsulated by connective tissue (Fig. 3b), and (ii) very late stages of atresia (gamma/delta) (Fig. 3c–f) which are yellow-orange-brown in colour and often referred to as melano-macrophage centres or brown bodies [35], [36], [37].

The maturity status and reproductive class of each female was determined using histological analysis (see Table 2) rather than macroscopic staging methods which can have a high rate of misclassification, particularly in regenerating females. Females were classified as mature if they had yolked oocytes (advanced, migratory nucleus or hydrated), atresia of yolked oocytes (alpha or beta stage) and/or maturity markers in the ovary. Immature fish were those with unyolked or early yolked oocytes in the ovary (Schaefer, 1998; Itano, 2000; Sun et al., 2010) and no atresia or maturity markers. All histological sections were read at least twice before a reproductive class was assigned.

**Table 2:** Histological classification criteria for South Pacific albacore.

Class	Maturity status	Activity	Development class	MAGO and POF stage	α and β atresia of yolked oocytes	Maturity markers
1	Immature	Inactive	Immature	Unyolked, no POFs	Absent	None
2	Immature	Inactive	Developing	Early yolked, no POFs	Absent	None
3	Mature	Active	Spawning capable	Advanced yolked, no POFs	<50% α atresia, β atresia may be present	May be present
4	Mature	Active	Spawning	Migratory nucleus or hydrated and/or POF's	<50% α atresia, β atresia may be present	May be present
5	Mature	Inactive	Regressing - potentially reproductive	Advanced yolked, no POFs	≥50% α atresia, β atresia present	May be present
6a	Mature	Inactive	Regressed 1	Unyolked or early yolked, no POFs	100% α atresia, β atresia may be present	May be present
6b	Mature	Inactive	Regressed 2	Unyolked or early yolked, no POFs	No α atresia, β atresia present	May be present
7	Mature	Inactive	Regenerating	Unyolked or early yolked, no POFs	Absent	Present

MAGO  =  most advanced group of oocytes, POF  =  postovulatory follicle. Maturity markers include gamma and delta stages of atresia (brown bodies) and/or residual (unovulated) hydrated oocytes.

### Oocyte diameter

To estimate the variation in the sizes of the most advanced group of oocytes, 174 sections were randomly selected from ovaries with advanced yolked or migratory nucleus oocytes. For each section, the diameters of five oocytes in the MAGO were measured using an Olympus F view II digital camera mounted on the Leica Wild stereo microscope and AnalySIS 3.2 software, and the mean oocyte diameter calculated for each ovary.

### Spawning frequency and fecundity

Spawning frequency of females was estimated by the postovulatory follicle method of [38]. This method uses the incidence of mature females with postovulatory follicles less than 24 hours old to estimate the fraction of the population spawning per day (spawning fraction), which is then converted into spawning frequency (inverse of spawning fraction). The daily cycle of oocyte maturation, spawning time, and POF degeneration rate were examined using ovaries obtained from 71 females sampled immediately after death in the Cook Islands and American Samoa. The time of death was compared to the MAGO present in the ovary and the age assigned to POFs.

Batch fecundity was estimated by the gravimetric method [39] for females with late stage migratory nucleus or hydrated oocytes. For each fish, a core subsample of between 0.05–0.09 g was taken from the middle region of both ovary lobes, weighed to the nearest 0.01 mg, and fixed in 10% buffered formalin. Each subsample was teased apart and the number of migratory nucleus or hydrated oocytes was counted under a Wild M5a stereomicroscope. The number of oocytes per weight of the subsample was raised to the weight of the ovary lobe to give an estimate of batch fecundity for the lobe. Estimates for the two lobes were summed to give a batch fecundity estimate for the fish. Fecundity for the female population by length, age and month was estimated as the product of batch fecundity at length and age, and spawning fraction at length and age in each month. These were then summed across months to estimate the potential annual fecundity by length and age for albacore.

### Statistical analyses

Generalized linear mixed-effects models (GLMM, implemented in R package lme4: [40]) were used to examine the effects of *FL*, month, longitude and latitude on the sex ratio, *GI*, oocyte diameter, proportion of active females, spawning fraction and batch fecundity. MAGO stage was included as an additional term in the analysis of oocyte diameter since it was expected that oocyte diameter would increase with MAGO stage. In addition, we tested for the effects of age on the spawning fraction and batch fecundity. For the fixed effects, we modelled *FL*, age, latitude, longitude and month as factors and cubic splines with varying degrees of freedom, and MAGO stage as a factor only. We modelled each factor as an additive term and as interactions with other factors. The factor fishing set was modelled as a random effect term in all models because multiple individual fish were often sampled at the same time from a single location and therefore fish within a set were not independent. The response variables sex ratio, proportion of active females and spawning fraction were modelled with a binomial distribution and logit link function, while GI, oocyte diameter and batch fecundity were modelled using a Gaussian distribution. For each response variable, Akaike's Information Criteria for small sample sizes (AIC_c_:[41]) was used to determine the level of support for including each of the explanatory variables in the models and to compare functional forms for the relationship between factors and dependent variables. Models with AIC_c_ values within two of that calculated for the best approximating model (lowest AIC_c_) were considered to describe the data equally well. The best model was then used to predict expected values of the response variable across the observed ranges of the informative explanatory variables. The Kolmogorov–Smirnov test was applied for normality of relative fecundity data.

## Results

### Sex ratio

**Figure 4:**
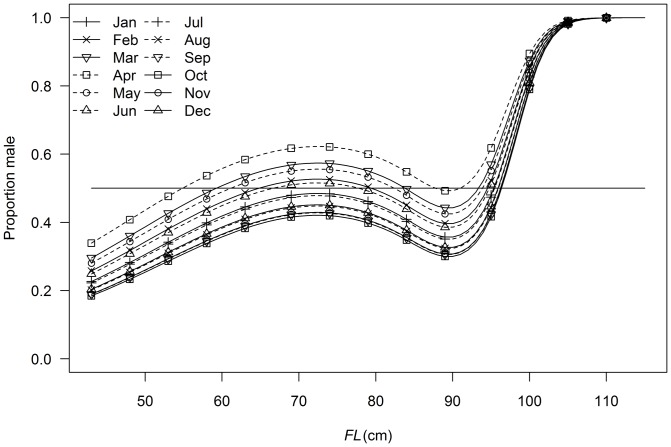
Predicted trends in sex ratio (proportion male) with fork length (*FL*) and month. Predictions derived from best fit model described in Table 3.

Variation in sex ratio was best described by a model that included terms for *FL* and month, as indicated by the model with the lowest AIC_c_ value (Table 3). This model indicated that sex ratio was female-biased for fish less than approximately 60 cm *FL* and between 85 and 95 cm, close to 1:1 between approximately 60 and 85 cm *FL*, and became progressively more male-biased for fish above 95 cm *FL* (Fig. 4). This pattern was consistent across months (Fig. 4). The mean sex ratios were more male-biased from January to June than from July to December in all *FL* classes (Fig. 4).

**Table 3:** Parameter estimates from generalized linear mixed-effects models (GLMM).

Analysis	Model	AIC_c_	ΔAIC_c_	*w*
Sex ratio	*P*_M_ = *s*(*FL*, df = 3) + *s*(*M*, *df* = 2) + *β*_set_ + *ε*	4078.80	0	0.98
	*P*_M_ = *s*(*FL*, *df* = 3) + *β*_set_ + *ε*	4088.95	10.14	0.01
Oocyte diameter	*O* = *s*(*FL*, *df* = 3) + *s*(*M*, *df* = 2) + MAGO + *β*_set_ + *ε*	1702.92	0	0.74
	*O* = *s*(*FL*, *df* = 2) + *s*(*M*, *df* = 2) + *lat* + *lon* + MAGO + *β*_set_ + *ε*	1706.92	4.01	0.10
	*O* = *s*(*FL*, *df* = 2) + *s*(*M*, *df* = 2) + *lat* + MAGO + *β*_set_ + *ε*	1707.35	4.44	0.08
Gonad index (Females)	*GI* = *s*(*lon*, *df* = 2) + *s*(*M*, *df* = 2) + *β*_set_ + *ε*	1646.97	0	1.00
Gonad index (Males)	*GI* = *s*(*lon*, *df* = 2) + *s*(*M*, *df* = 2) + *β*_set_ + *ε*	1183.46	0	1.00
Active females(*FL*)	*P*_A_ = *s*(*FL*, *df* = 2) + *s*(*M*, *df* = 2) + *β*_set_ + *ε*	301.15	0	0.51
	*P*_A_ = *s*(*FL*, df = 2) + *s*(*M*, df = 2) + *s*(*lat*, df = 2) + *s*(*lon*, df = 2) + *β*_set_ + *ε*	302.47	1.31	0.26
	*P*_A_ = *s*(*FL*, *df* = 2) + *s*(*M*, *df* = 2) + *lat* + *β*_set_ + *ε*	302.75	1.60	0.23
Active females(*A*)	*P*_A_ = *A* + *s*(*M*, *df* = 2) + *β*_set_ + *ε*	194.56	0	0.55
	*P*_A_ = *A* + *s*(*M*, *df* = 2) + *lat* + *β*_set_ + *ε*	196.62	2.06	0.20
	*P*_A_ = *A* + *s*(*M*, *df* = 2) + *lat* + *lon* + *β*_set_ + *ε*	196.72	2.16	0.19
	*P*_A_ = *s*(*M*, *df* = 2) + *lat* + *β*_set_ + *ε*	198.69	4.13	0.07
Spawning fraction (*FL*)	*P*_P_ = *FL* + *s*(*M*, *df* = 5) + *β*_set_ + *ε*	565.69	0	0.60
	*P*_P_ = *s*(*M*, *df* = 4) + *lat* + *β*_set_ + *ε*	568.12	2.43	0.18
	*P*_P_ = *FL* + *s*(*M*, *df* = 2) + *lat* + *β*_set_ + *ε*	568.25	2.56	0.17
	*P*_P_ = *FL* + *s*(*M*, *df* = 2) + *lat* + *lon* + *β*_set_ + *ε*	570.25	4.57	0.06
Spawning fraction (*A*)	*P*_P_ = *A* + *s*(*M*, *df* = 4) + *β*_set_ + *ε*	366.85	0	0.43
	*P*_P_ = *A* + *s*(*M*, *df* = 2) + *lat* + *β*_set_ + *ε*	367.11	0.26	0.37
	*P*_P_ = *A* + *s*(*M*, *df* = 2) + *lat* + *lon* + *β*_set_ + *ε*	368.39	1.54	0.20
Batch fecundity (*FL*)	*B* = *s*(*FL*, *df* = 2) + *s*(*M*, *df* = 3) + *β*_set_ + *ε*	64.20	0	1.00
Batch fecundity (*A*)	*B* = *s*(*A*, *df* = 2) + *s*(*M*, *df* = 3) + *β*_set_ + *ε*	39.56	0	1.00

Models examine the effects of fork length (*FL*), age (*A*), month (*M*), latitude (*lat*), longitude (*lon*) and MAGO stage on the sex ratio (*P*_M_, probability of being male), gonad index (*GI*), oocyte diameter (*O*), proportion of active females (*P*_A_), spawning fraction (*P*_P_, proportion with POFs) and batch fecundity (*B*). Only models with at least 1% support (Akaike weight ≥0.01) are shown. The effects of *FL*, age and month were modelled as factors and cubic splines (*s*) with varying degrees of freedom (*df*). MAGO stage was modelled as a fixed factor only. Sex ratio, *P*_A_ and *P*_P_ were modelled with a binomial distribution and logit link function. *β*_set_ is the random effect of fishing set, and *ε* is the error term. AIC_c_ is the small-sample bias-corrected form of Akaike's information criterion, Δ is the Akaike difference, and *w* is the Akaike weight.

### Gonad development

**Figure 5:**
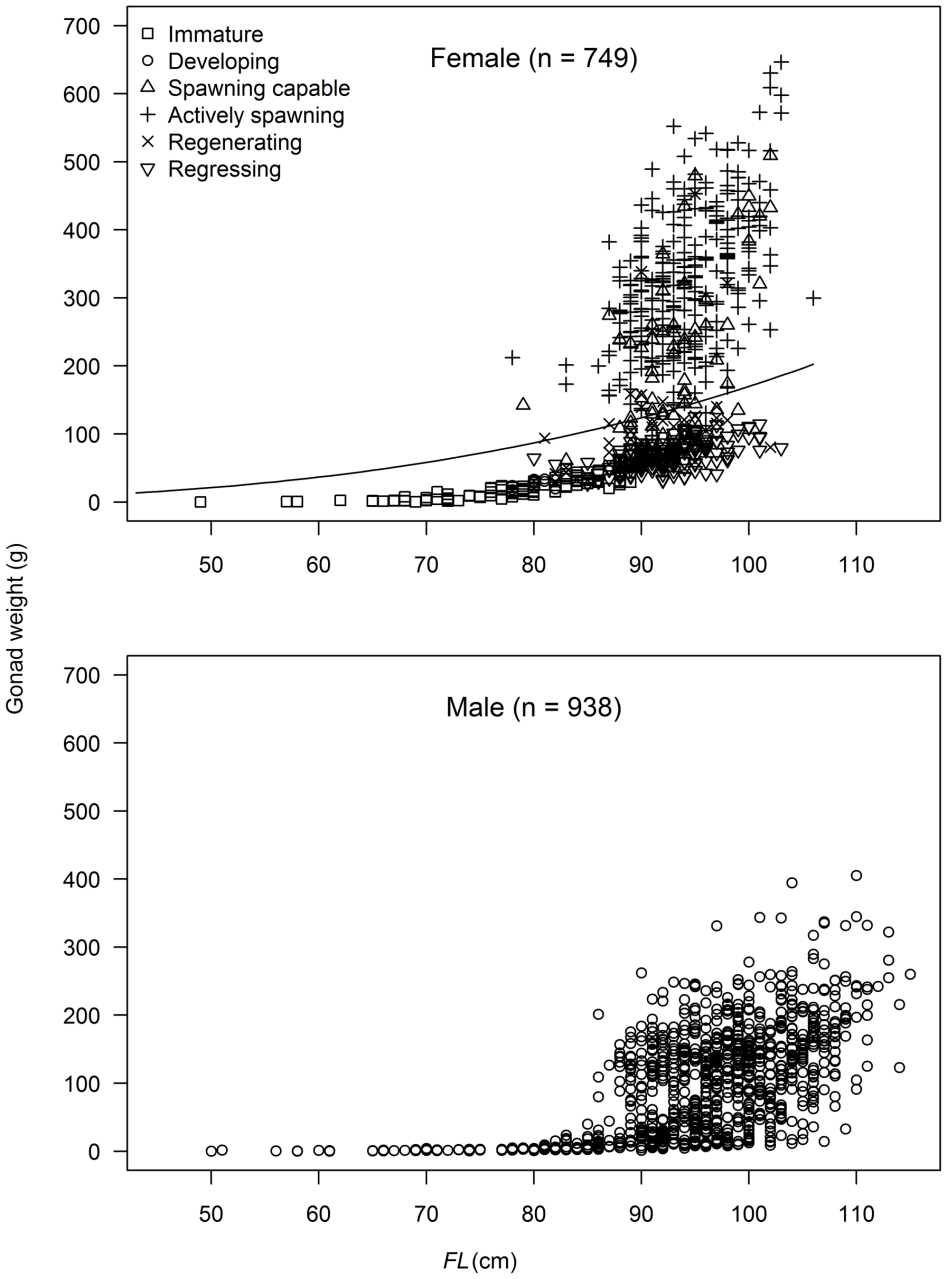
Observed gonad weight at fork length (*FL*) for male and female South Pacific albacore. Data are presented by reproductive stage for females (but not males) and the regressing class includes classes 5, 6a and 6b (see Table 2). The curve plotted for females represents a gonad index of 1.7, above which individuals are assumed to be mature [27].

Gonad weight (*GW*) was available for approximately half of the fish sampled. Of these, most fish <85 cm *FL* showed little gonad development (Fig. 5). Histological analysis indicated that females <85 cm *FL* were predominantly immature except for a small number of fish between 74 and 85 cm *FL* that were mature and classed as either spawning capable, spawning, regressing or regenerating (Fig. 5). Very few females classed as maturing (class 2) were sampled (n = 16) and all had ovaries weighing less than 60 g. These fish were caught year-round and at latitudes between 18°S and 27°S, and were considered immature following [28], [29], [30], [31]. The largest immature female was 94 cm *FL* while the smallest spawning female was 78 cm *FL*. All females with a *GI*>1.7, identified by [27] as the value above which females were mature, were indeed classed as mature and most were either spawning capable or spawning (Fig. 5). However, a proportion of females with a *GI*<1.7 were also mature (mostly regressing or regenerating). In the current study, all females with a GI<0.38 were classed immature and all with a GI>0.86 were mature.

**Figure 6:**
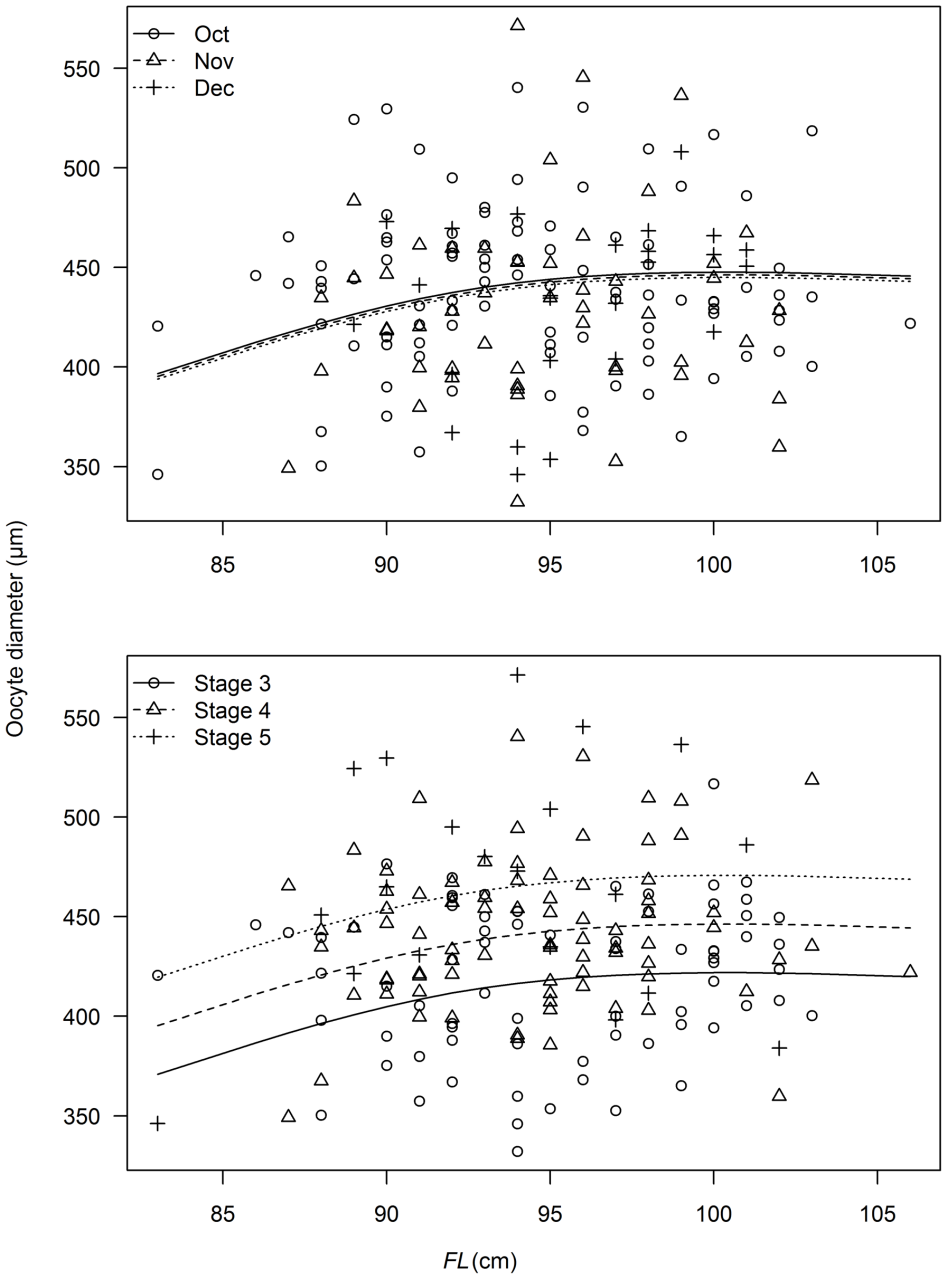
Observed oocyte diameter and predicted trends with fork length (*FL*), month and oocyte stage. Predictions derived from best fit model described in Table 3. Predicted oocyte diameter based on oocytes at stage 4 in upper panel and the month of November in lower panel. Data were available only for the peak spawning months of October, November and December.

Measurements of oocyte diameter were highly variable and ranged from 332 to 571 µm (Fig. 6). Variation in oocyte diameter was best described by a model that included terms for *FL*, MAGO stage and month. There was limited statistical support for also including latitude and longitude (Table 3). The best-fitting model indicated that oocyte diameter increased with *FL* as MAGO stage increased, and declined between October and December (Fig. 6).

### Spawning season and location

**Figure 7:**
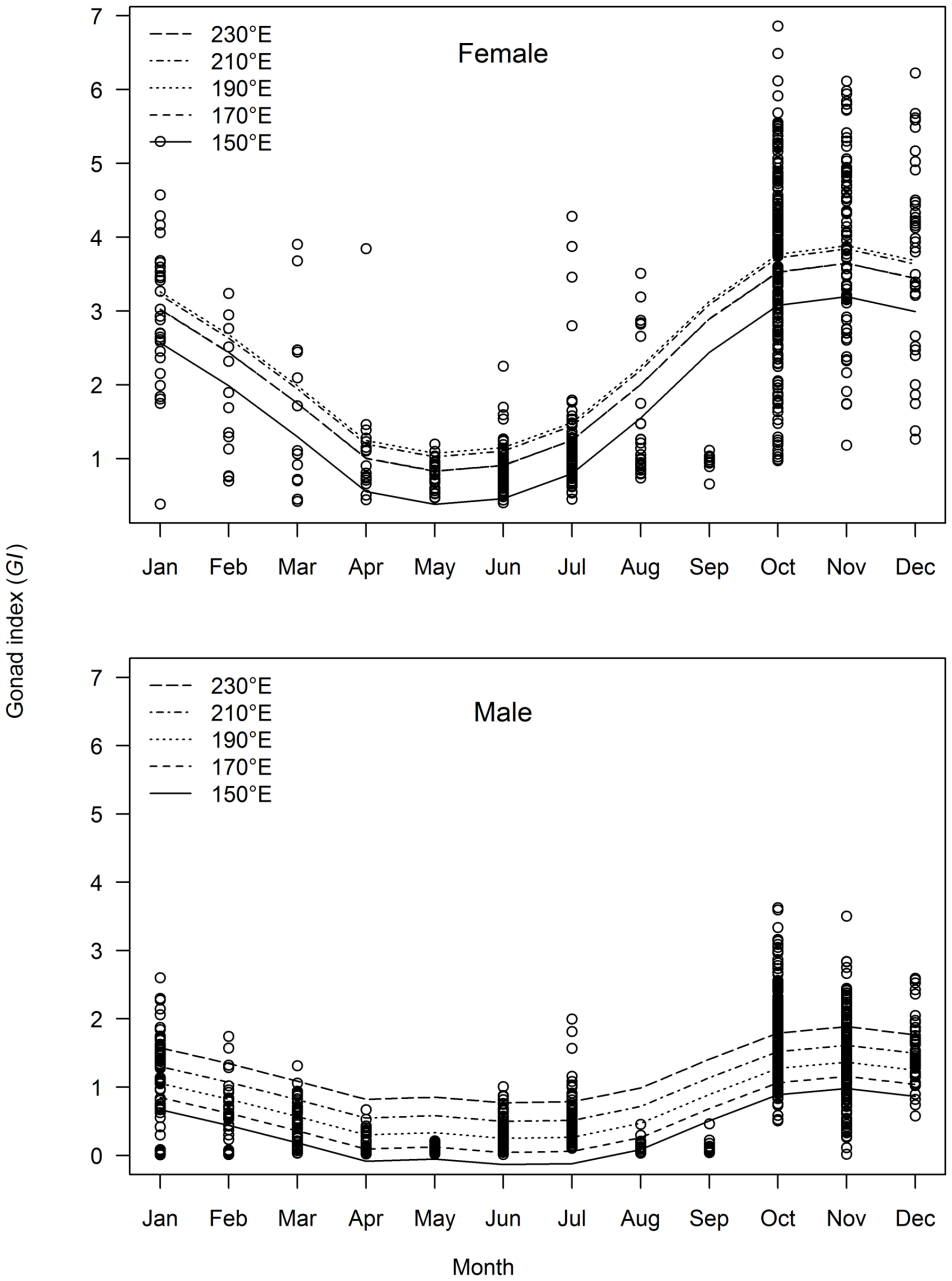
Gonad index and predicted trends with month and longitude for mature females and all males. Predictions derived from best fit model described in Table 3.

The gonad index (*GI*) was highly variable among individuals, particularly for females (Fig. 7). This variation was described best by a model that included terms for month and longitude for both mature females and males (Table 3). The seasonal patterns in monthly *GI* indicate that fish spawn during the austral spring and summer. For mature females, mean *GI* was highest between October and December, and declined from January to May (Fig. 7). Monthly patterns in mean *GI* for males were similar to females, with the highest values in October to January (Fig. 7). This seasonal pattern was consistent across longitudes, but mean *GI* increased with longitude for both females and males (Fig. 7).

**Figure 8:**
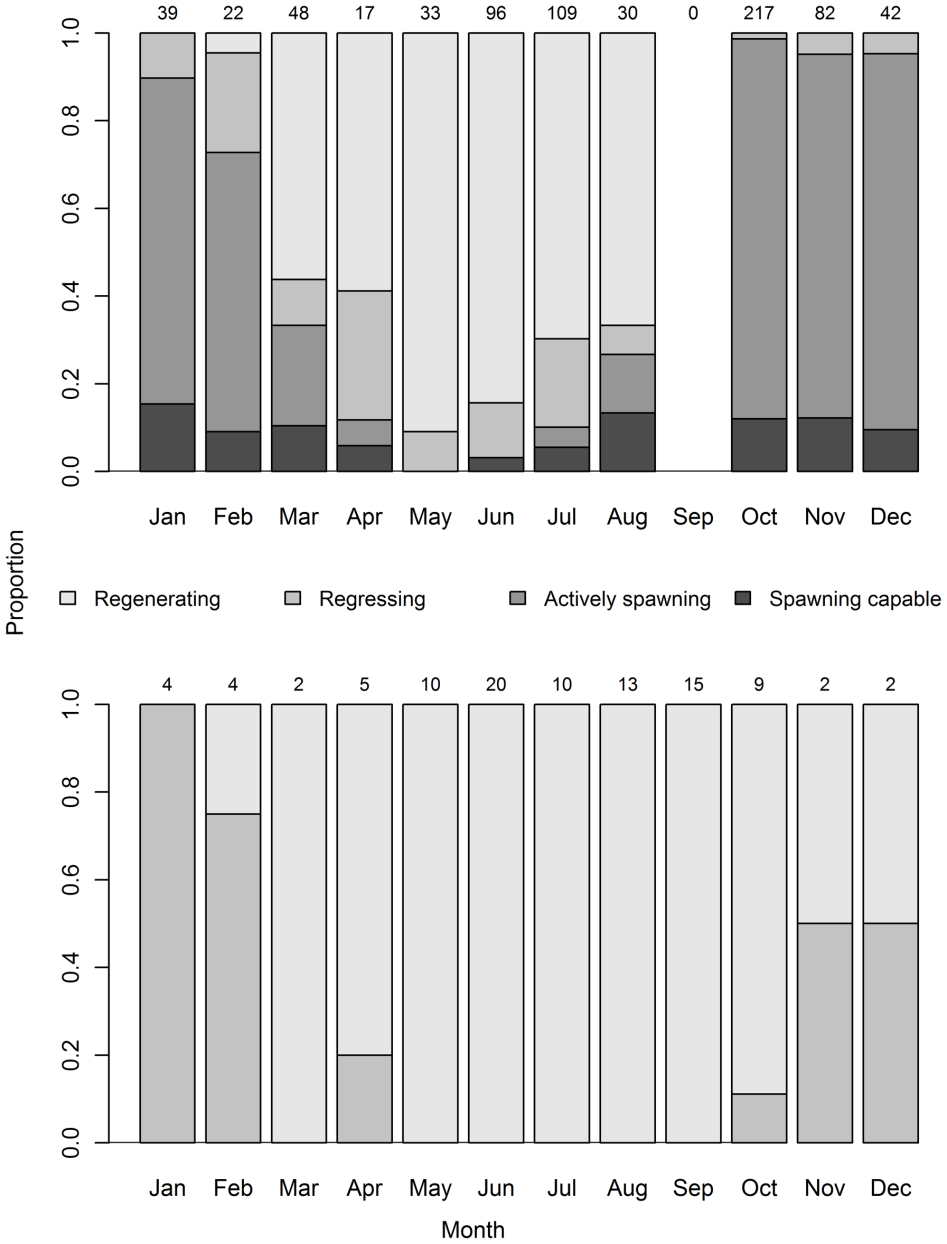
Proportion of development classes of mature female South Pacific albacore by month sampled. Top panel represent fish sampled north of 25°S and bottom panel represents fish sampled south of 25°S. The regressing class includes classes 5, 6a and 6b (see Table 2). Sample size per month shown at top of each bar.

Active females were caught in a broad area between approximately 10 and 25°S (Fig. 1). The seasonal pattern in frequency of development classes of mature females in these latitudes was consistent with the *GI* data (Fig. 8). Spawning and spawning capable females were sampled predominantly during the austral spring and summer months; their abundance was relatively low in July to August, high in October to December, and declining between January and April (Fig. 8). It is unknown whether significant levels of spawning also occur in September since no mature females were sampled north of 25°S in this month. Actively spawning females were caught at locations with SST's between 24 and 29 °C, with a mean 27.4 °C. Regressing females were present in relatively low numbers in all months of the spawning season, while the proportion of regenerating females increased after January and was the dominant stage during the autumn and winter period from March to August. All mature females sampled south of 25°S were either regressing or regenerating (Fig. 8) and their relative abundance was consistent with the spawning cycle: regressing females occurred during and immediately after the peak spawning months, while regenerating females were most abundant in the non-spawning season. Overall, very few mature fish were sampled south of 25°S during the spawning months, presumably because the majority were further north at spawning latitudes.

**Figure 9:**
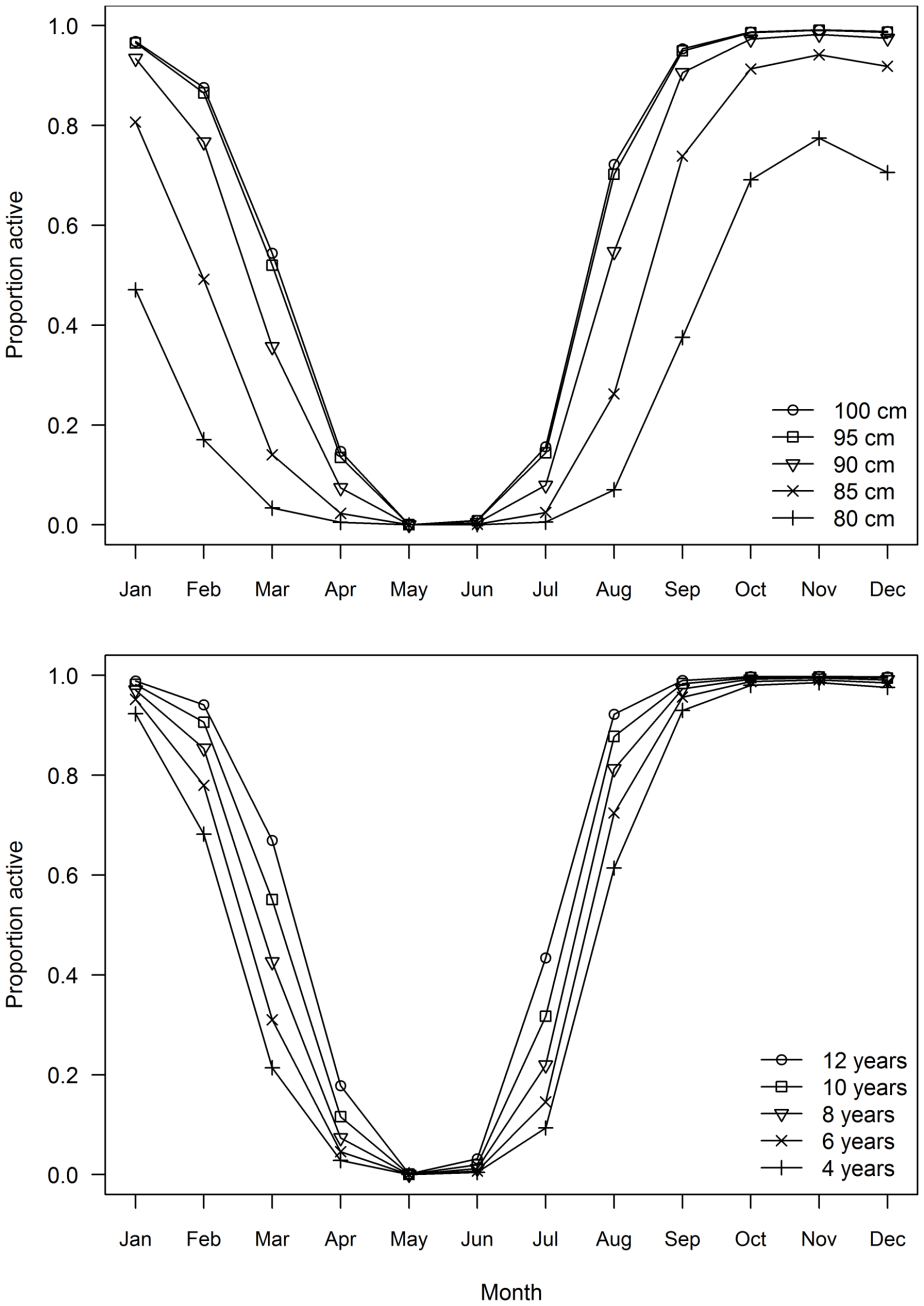
Predicted trends in the proportion of active females with month for South Pacific albacore. Top panel represent trends with fork length (*FL*) and bottom panel represents trends with age. Predictions derived from best fit model described in Table 3.

None of the GLMM models was unambiguously the best model to describe the variation in the proportion of mature females in an active state (spawning capable and spawning) as indicated by ΔAIC_c_ values <2 and Akaike weights between 0.23 and 0.51 for the three best-fitting models (Table 3). However, all models included terms for month and either length or age (Table 3). There was also some support for latitude and longitude effects. The best fitting models predicted that the proportion of active females was lowest between April and July and highest between October and January (Fig. 9). For each month, however, the proportion active was lower for small and young fish and increased with increasing fish length and age (Fig. 9). Small and young fish were relatively inactive at both the start and end of the spawning season compared to larger and older fish, and a smaller proportion were active during the peak spawning months of October to December.

### Time of spawning and POF degeneration

**Figure 10:**
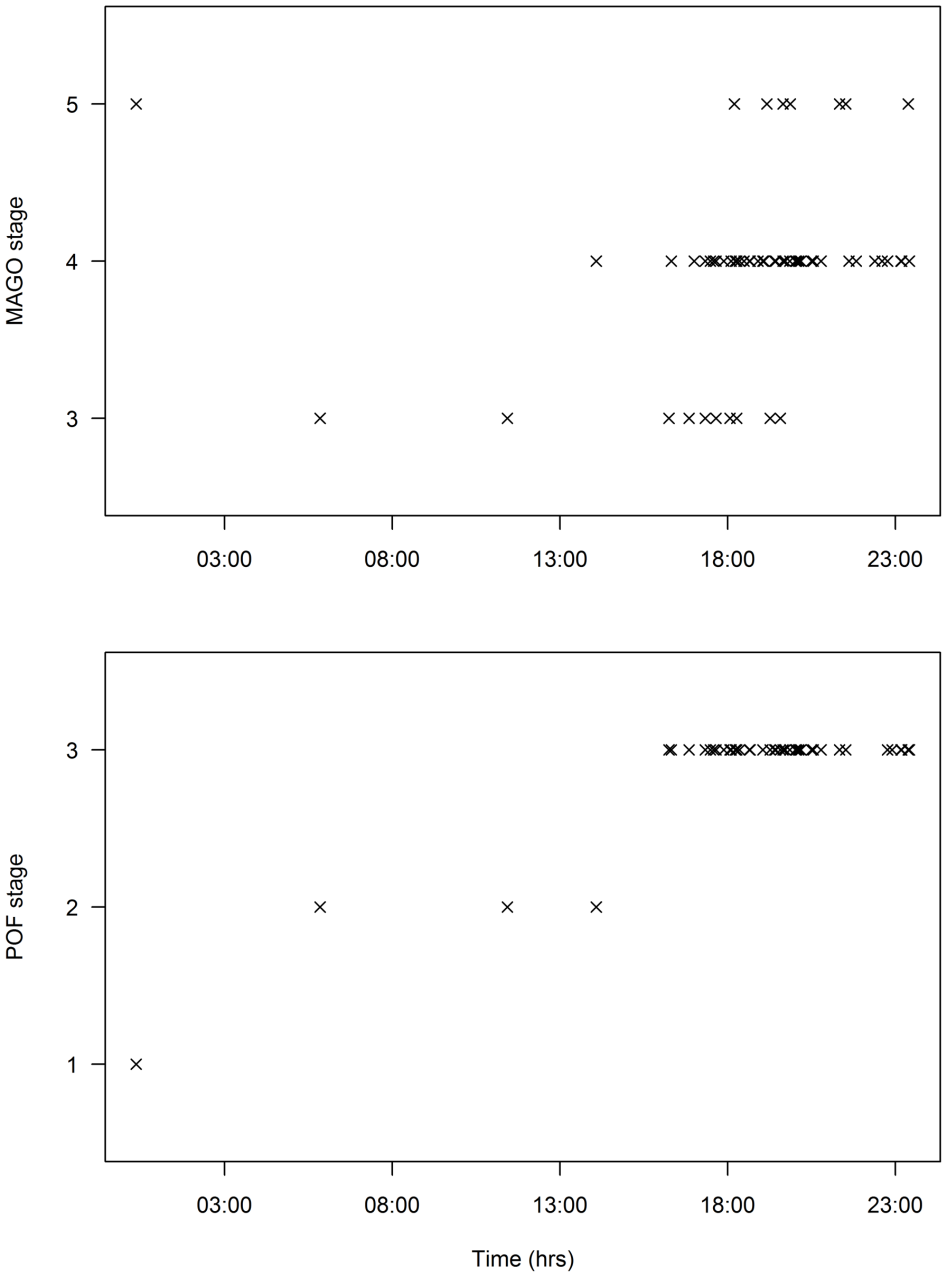
Most advanced oocyte stage (upper) and postovulatory follicle stage (lower) at the time of death. Most advanced oocyte (MAGO) stages are advanced yolked (3), migratory nucleus (4), and hydrated (5). POF stages are new (1), <12 hours (2), and >12 hours (3). POF  =  postovulatory follicle.

The majority of females sampled immediately after death were landed between 1600 hrs and midnight, although a few were also landed earlier in the day (Fig. 10). Of the females classed as spawning (n  =  58), advanced yolked oocytes were apparent from 0551 until 1834 hrs (mean 1603 hrs), migratory nucleus oocytes from 1405 until 2325 hrs (mean 1933 hrs) and hydrated oocytes from 1812 until 0022 hrs (mean 2056 hrs) (Fig. 10). This suggests that spawning is synchronized in albacore and oocyte maturation is complete within 24 hours.

The ages assigned to POFs were consistent with a 24 hour cycle of degeneration in albacore. Only one fish had stage 1 POFs and this fish was sampled just after midnight (Fig. 10). The ovary also contained hydrated oocytes indicating that the fish was landed as it was about to spawn or that the fishing operation/sampling caused hydrated oocytes to be released. The absence of stage 1 POFs prior to midnight confirms that albacore spawn in the early hours of the morning. Although the sample size was low for the hours between 1 am and 4 pm, POFs became progressively older during the day and all POFs >12 hours old were sampled after 1400 hrs (Fig. 10). No ovaries contained POFs of different ages.

### Spawning frequency

Of the mature fish sampled between 10 and of 25°S, the fraction with POFs was 0.45, resulting in an estimated mean annual spawning interval of 2.2 days. During the peak spawning months of October to December, however, the spawning fraction was 0.75 resulting in an estimated mean spawning interval of 1.3 days. In these months, the ovaries of 87.7% of active females contained evidence of recent or imminent spawning activity, and 52.7% contained evidence of two spawning events (postovulatory follicles and either migratory nucleus or hydrated oocytes) indicating that albacore is capable of spawning daily.

**Figure 11:**
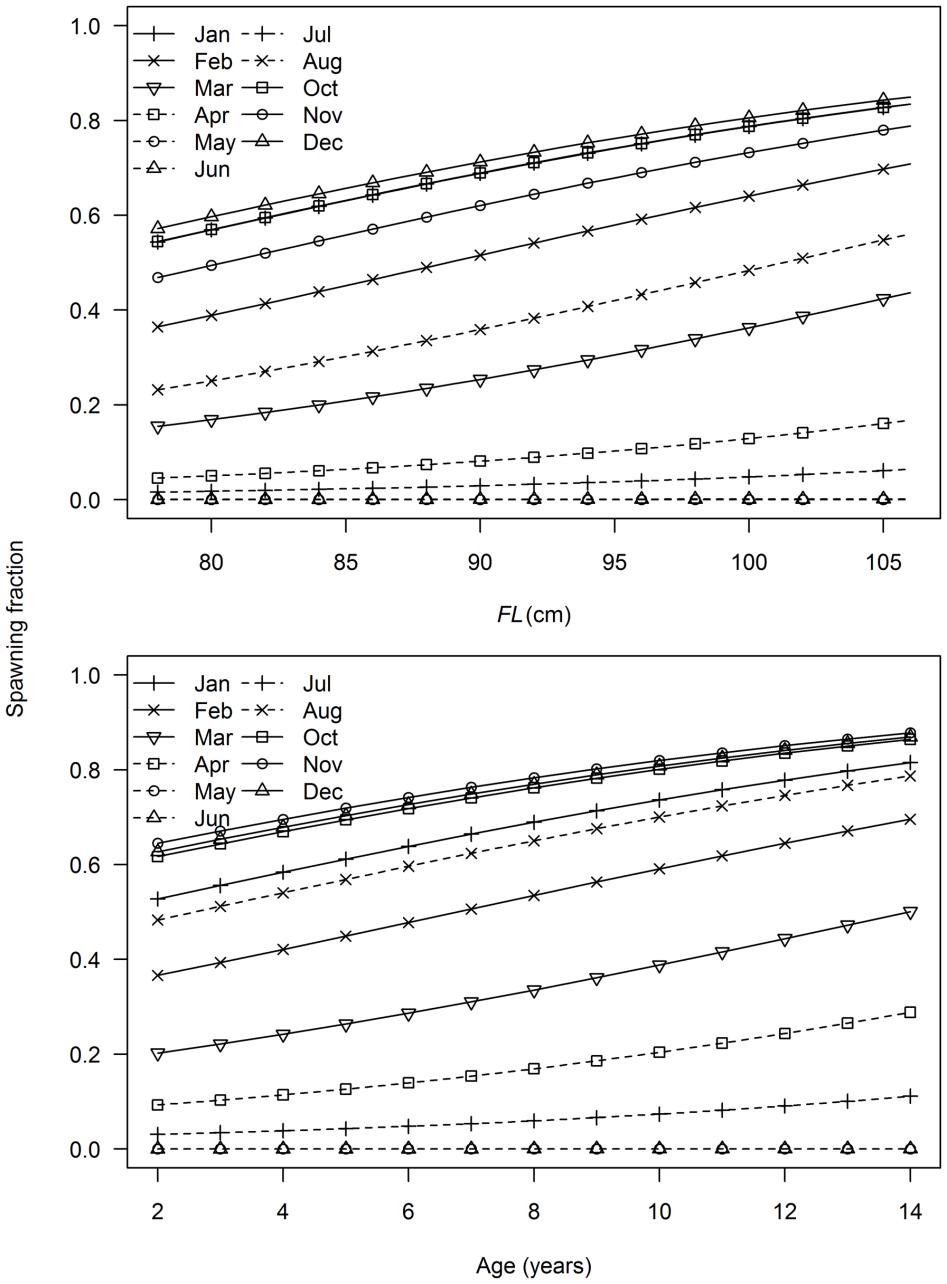
Predicted trends in spawning fraction with fork length (*FL*), age and month. Predictions derived from best fit model described in Table 3. No active females were sampled in September.

Variation in spawning fraction (proportion of mature females with POFs) was described best by a model that included terms for length and month, but none of the GLMM models was unambiguously the best model to describe the variation in spawning fraction with age as indicated by ΔAIC_c_ values <2 and Akaike weights between 0.20 and 0.43 for the three best-fitting models (Table 3). There was support for both latitude and longitude effects on the variation in spawning fraction with age (Table 3).The best-fit model predicted that spawning fraction increased with length and age, and was lowest between April and July and highest between October and January (Fig. 11).

### Batch and potential annual fecundity

Of the ovaries sampled whole, 71 contained hydrated oocytes (with no new POFs) or late stage migratory nucleus oocytes suitable for batch fecundity estimation. All of these ovaries were collected in the spawning months of October to March, except two individuals collected in July. Batch fecundity ranged from 0.26 million oocytes for a 97 cm female sampled in Fiji to 2.83 million oocytes for a 98 cm female sampled in Tonga (mean ± SD was 1.20 ± 0.50). The relative batch fecundity ranged from 13.2 to 137.2 oocytes per gram of body weight (mean ± SD was 64.4 ± 24.7) with a normal distribution.

**Figure 12:**
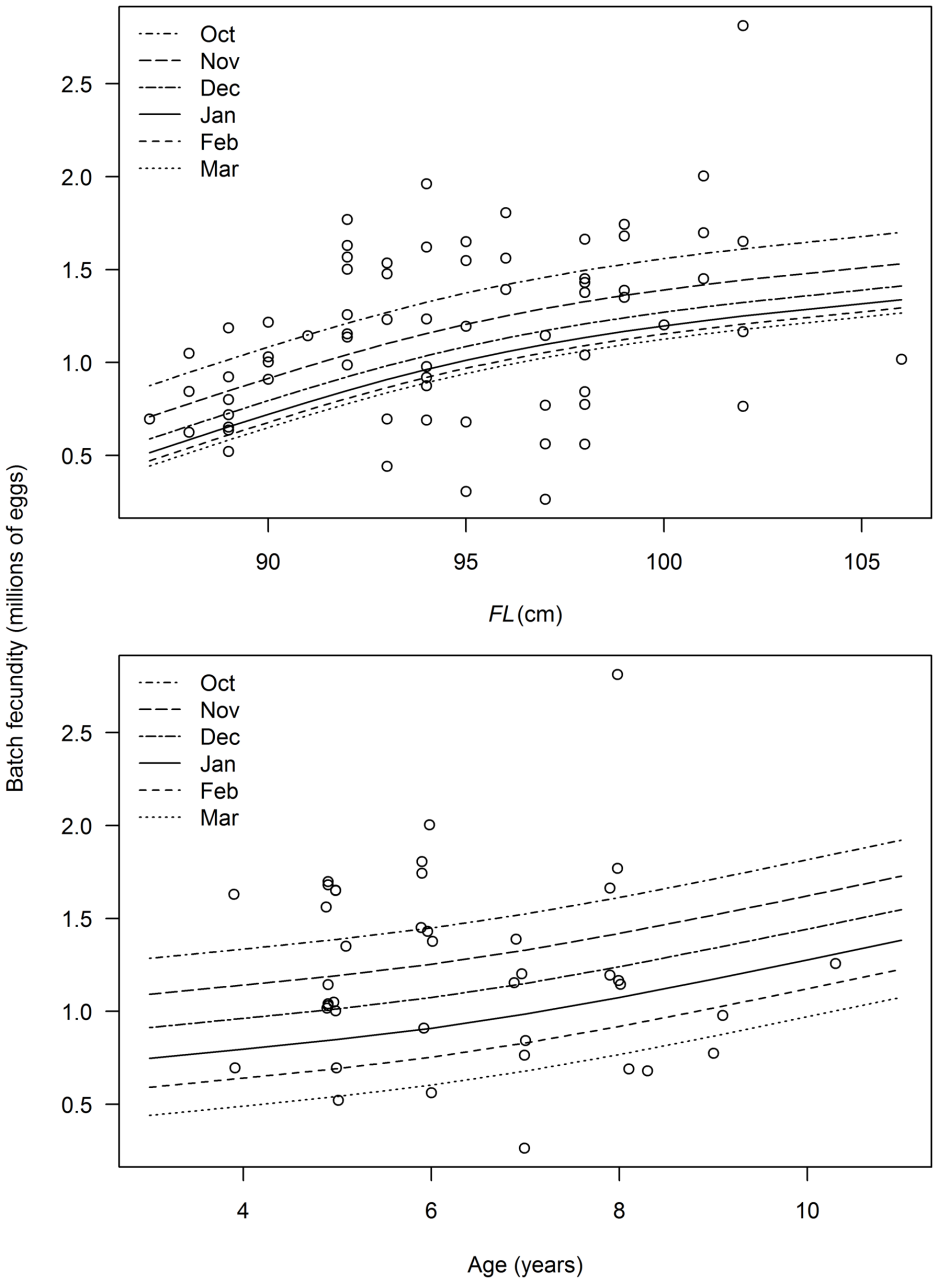
Estimated batch fecundity and predicted trends with fork length (*FL*), age and month. Predictions derived from best fit model described in Table 3.

**Figure 13:**
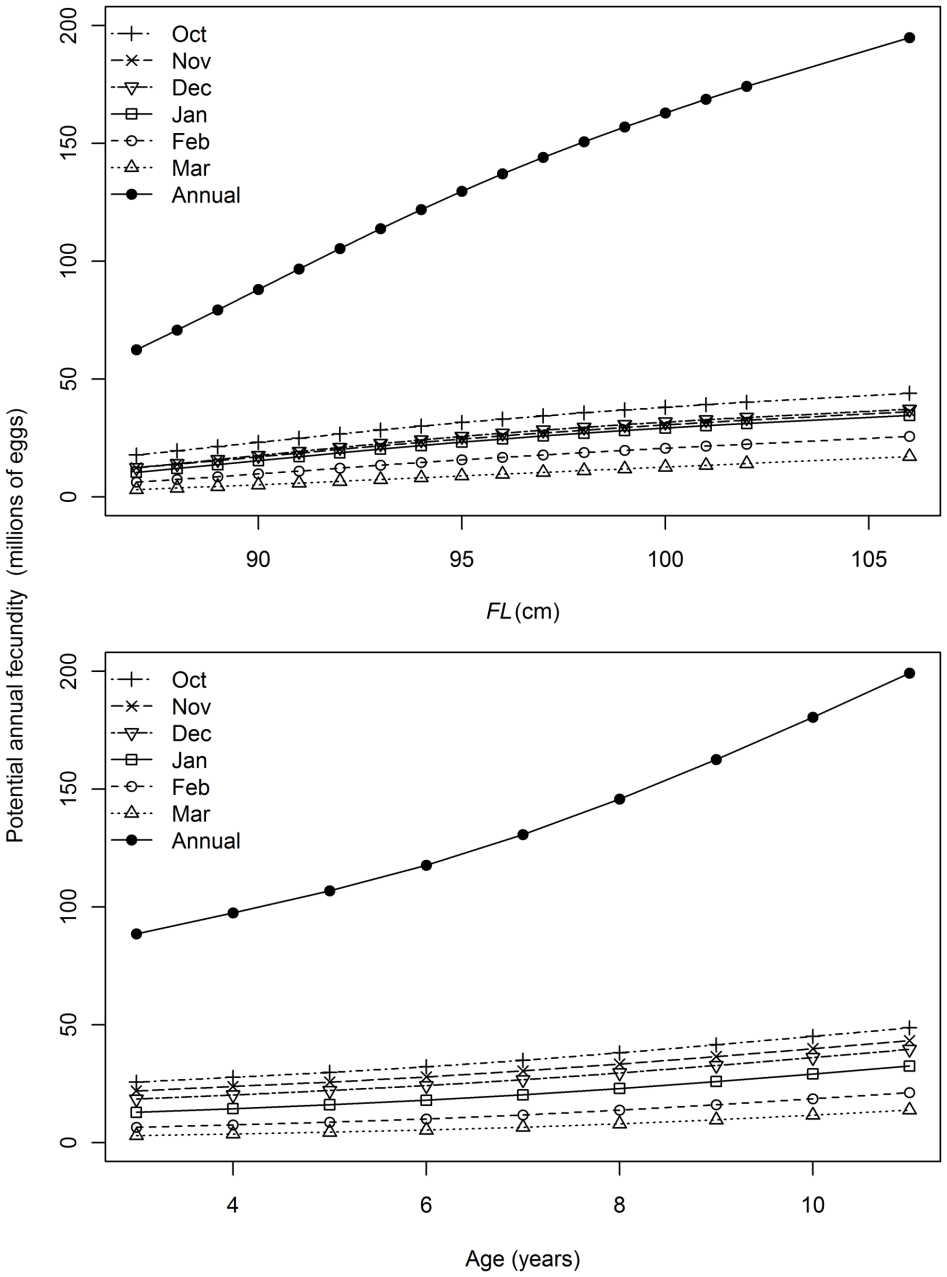
Estimated trends in potential monthly and annual fecundity with fork length (*FL*) and age.

Models that included terms for month, length and age best described the variation in batch fecundity (Table 3). Predicted batch fecundity increased with length and age and was higher between October and December than between January and March (Fig. 12). Predicted potential monthly and annual fecundity increased with length and age (Fig. 13). Potential annual fecundity in millions of eggs per year ranged from 62.4 for an average 87 cm female to 194.8 for an average 106 cm female. Over the range of ages sampled, potential annual fecundity ranged from 88.6 for a 3 year-old to 199.3 for an 11 year-old.

## Discussion

This study applied consistent analytical methods to data obtained from samples of albacore collected throughout the South Pacific Ocean. This approach avoids study fragmentation, which is a key problem limiting the understanding of many widespread species, including most tunas. Spawning activity for albacore appears to be synchronized across the entire South Pacific Ocean (peaking in October to December) and length/age and/or season have important effects on many reproductive parameters including sex ratio, gonad development, spawning season and location, spawning frequency and batch fecundity. Spatial differences in gonad development at a regional scale were detected which further highlighted the importance of ocean-scale sampling programs to examine the life-history parameters of species with broad geographic distributions.

Analysis of sex ratios indicated that males dominate length classes above 95 cm *FL*. A preponderance of males in larger size classes has been found in several tuna species including southern bluefin, Atlantic bluefin, bigeye, yellowfin as well as albacore [14], and it has been suggested that this dominance could be due to sex related differences in availability, growth rates, or natural mortality [42]. Differences in natural mortality have to date been generally accepted as the most likely cause [14], [43], with higher mortality in females possibly due to greater costs associated with spawning [29]. However, differential growth rates between the sexes have recently been demonstrated by [23], who found that male South Pacific albacore reach significantly larger asymptotic size than females. In addition, the current work found an accumulation of females between 85 and 95 cm, which are the length classes preceding those with a male bias. Such an accumulation of females is expected if sex-specific growth is responsible for the observed changes in sex ratio [42]. While ontogenetic shifts in availability or mortality cannot be unequivocally excluded, these observations are consistent with female albacore diverting more resources than males to reproduction rather than somatic growth following sexual maturity. Male bias was also greater from February to June coinciding with the end of the peak in spawning activities suggesting that sex differences in movement and/or seasonal changes in sex-specific selectivity may also contribute to the patterns observed.

Albacore in spawning condition were sampled between 10 and 25°S. The seasonal changes in *GI* were consistent across longitudes and sexes, with the highest values occurring during spring and summer months. Histological analysis of ovaries confirmed that peak spawning activity occurred during October to December, although spawning females were caught in all months of the year except May and June. Spawning activity occurred in areas where surface temperatures exceeded 24 °C, which is consistent with temperatures at which spawning occurs in other tunas [14]. The timing of peak spawning activity is also consistent with the southern movement of the 24 °C isotherm into subtropical latitudes, bringing spawning habitat into the distributional range of albacore [14]. Our results are similar to previous studies showing that spawning in the central South Pacific occurs between September/October and February/March, peaking in December [27], [44]. In the northeast Pacific Ocean, albacore also spawns over an extended spring/summer period (March to September) between 10–20°N [28], although larval surveys suggest that some spawning also occurs during winter and as far north as 30°N [13]. Year-round spawning in the South Pacific as suggested by [45] was not confirmed, although additional sampling would be required to confirm spatiotemporal patterns of albacore spawning north of 10°S.

Evidence of regional variation in *GI*, with fish in easterly longitudes on average having heavier gonads for their size than fish further west, has not been reported previously in other tunas. This variation may result from small longitudinal effects combined across several parameters. Samples were not collected in the east over the whole spawning season which may have reduced our ability to detect regional differences. The greater investment in ovary development for eastern fish is consistent with faster growth rates also observed in these fish [23].

Like other tunas, albacore has asynchronous oocyte development and is a multiple spawner with indeterminate annual fecundity [46]. Our results are consistent with albacore being nocturnal spawners with spawning activity synchronized during the early hours of the morning. Nocturnal spawning has been found in yellowfin, Atlantic bluefin, and bigeye and in many other fish species [14], [47]. It is important to know the length of time that POFs remain visible in ovaries after spawning to estimate spawning frequency using the POF method [39]. The maximum duration of detection for POFs in skipjack tuna (*Katsuwonus pelamis*) and yellowfin tuna is about 24 hours after spawning [29], [33], [48]. The current study confirms that albacore also has a 24 hour cycle for POF degeneration. Similar POF resorption rates for skipjack, yellowfin and albacore tuna are not surprising given that they spawn when surface temperatures are above 24 °C [14] and water temperature is assumed to be the dominant factor determining POF resorption rates in fish [49]. However, albacore are thought to spawn at depths of ∼100–200 m where temperatures are cooler than at the surface [18]. Since albacore are capable of regulating their body temperature [50], it may be internal temperature rather than water temperature that directly influences the rate of POF resorption in tuna.

The mean spawning interval of 1.3 days obtained for albacore in October to December is lower than the 1.7 days found in a recent study in the North Pacific during peak spawning in April [28]. Albacore are capable of spawning daily since the ovaries of over 50% of active females sampled in the peak spawning months contained evidence of both recent (POFs) and imminent spawning (migratory nucleus or hydrated stage oocytes). Spawning interval has not been estimated for albacore in other oceans, but estimates for other tuna species range from 1.1 for bigeye to 1.6 for southern bluefin, with almost daily spawning estimated for the latter when active [14] [32].

The presence of regressing or regenerating females during the spawning season suggests that some fish may not spawn for the entire period, or at all in some years. The duration of spawning activity by individuals could not be determined directly, but appears to be related to fish size and age. For all length and age classes, the proportion active and the spawning fraction were low at the start of the spawning season, highest from October to January, and low again at the end of the season. Lower activity at the start and end of a spawning season is expected as the proportion of fish spawning increases slowly and then declines at the end of the season. A low spawning fraction at the start of the spawning season has been observed in southern bluefin tuna [51]. In this case it was attributed to delayed spawning by some females after arriving on the spawning ground; this delay is thought to allow them to recover from their long migration from the southern oceans to the northeast Indian Ocean spawning area. We also found that the proportion active and spawning fraction were lowest in small/young fish each month, and increased with increasing fish size and age. Increasing spawning fraction with fish size has also been found in yellowfin and southern bluefin tuna [29], [51]. Our results are consistent with smaller and younger females having a shorter spawning season on average than larger, older fish. A low spawning fraction in small/young albacore is not surprising given that many are probably maturing and adjusting to tropical waters for the first time. A shorter season for small females may be related to smaller energy reserves, and the need to balance their reproductive investment with somatic growth and future spawning success. A shorter spawning season for smaller/younger fish has not been reported previously for tunas, but has been recognised in other pelagic species including jack mackerel (*Trachurus symmetricus*) and Pacific mackerel (*Scomber japonicus*) [52], [53]. An earlier start and delayed cessation of spawning has been recorded in several multiple spawners (see [54]).

The relationship between batch fecundity and albacore length or age was highly variable, although significant positive relationships were predicted for each month. High variability in batch fecundity with length has been observed in several tuna species [29], [32], [34], [55], [56] and may be related to the stage in the spawning cycle when the fish were caught [32]. This is supported by the relationship found between batch fecundity and spawning month; albacore produce larger batches on average at the start of the spawning season (October to December) and smaller batches as the season progresses. Although there have been no direct observations of this previously in tunas, a decrease in batch fecundity has been found in Atlantic mackerel (*Scomber scombrus*) as fish migrated north during the spawning season [57]. Our estimate of mean relative batch fecundity for albacore of 64 (± 25) oocytes per gram of body weight is not significantly higher than in North Pacific albacore (50 oocytes per gram of body weight) [28], and is similar to yellowfin tuna in the eastern and western Pacific (62–68) [29], [58], [59], bigeye tuna in the western Pacific (60) [60] and southern bluefin tuna (57) [32]. Lower mean relative batch fecundity was reported for bigeye in the eastern and central Pacific Ocean (24–31) [14], [31].

Potential annual fecundity for indeterminate spawners requires estimates of batch fecundity, spawning fraction and the length of the spawning season [39], [61]. In tuna, annual fecundity has only been estimated for yellowfin tuna which spawn year-round in tropical waters and seasonally in sub-tropical waters when temperature are suitable [14], [29]. Annual fecundity was estimated at 223 × 10^6^ oocytes for a two year-old and 671 × 10^6^ oocytes for a three year-old [29]. It is more difficult to estimate annual fecundity for an indeterminate species which migrates to specific latitudes to spawn, such as albacore, as the duration of spawning and spawning frequency by individuals can be difficult to determine [61]. However, the current study shows that all reproductive stages of albacore are present on the spawning grounds during and after the spawning season, and potential annual fecundity can be estimated if sampling is undertaken at the appropriate spatial and temporal scale. The large spawning area and protracted spawning season makes short-term sampling in only a small number of areas potentially misleading in terms of estimating spawning potential.

The current stock assessment model uses sex ratio, proportion mature, spawning frequency and fecundity to estimate relative reproductive output [10], [21]. Further improvements in the assessment model would include accounting for the different spawning behaviour and reproductive output between the smallest/youngest and largest/oldest mature fish. In addition to significantly improving the understanding of the reproductive dynamics of albacore, the current study has provided an extensive set of estimates (including temporal, spatial, length and age effects) of these reproductive parameters for use in future stock assessments. The improved estimation of reproductive potential is likely to result in better generation of advice to develop management strategies to maintain reproductive potential above sustainability limits for albacore.
